# Editorial: Innate Immunity in Early Diverging Metazoans

**DOI:** 10.3389/fimmu.2022.816550

**Published:** 2022-01-31

**Authors:** Nikki Traylor-Knowles, William E. Browne, Laura D. Mydlarz, Caroline V. Palmer, Benyamin Rosental

**Affiliations:** ^1^ Department of Marine Biology and Ecology, University of Miami Rosenstiel School of Marine and Atmospheric Sciences, Miami, FL, United States; ^2^ Department of Biology, University of Miami, Cox Science Building, Miami, FL, United States; ^3^ Department of Biology, University of Texas Arlington, Arlington, TX, United States; ^4^ Flourish Editing, Devon, United Kingdom; ^5^ The Shraga Segal Department of Microbiology, Immunology, and Genetics, Faculty of Health Sciences, Regenerative Medicine and Stem Cell Research Center, Ben-Gurion University of the Negev, Beer Sheva, Israel

**Keywords:** invertebrate, innate immunity, metazoa, Cnidaria, Porifera

This Research Topic on innate immunity was motivated by the need to expand our knowledge of innate immunity and general immune responses in early diverging metazoans, such as cnidarians, placozoans, sponges, and ctenophores (**Figure 1**). Previous immunological studies in vertebrates and invertebrates, such as *Drosophila, C. elegan* and shellfish (1–5), launched the field of comparative invertebrate immunology. However, as this field flourished there remained an unbalanced focus on bilaterian models, limiting a broader understanding of the evolution and diversity of metazoan innate immune processes. Throughout the Metazoa there are undoubtedly shared core mechanisms of immunity, as well as uniquely evolved lineage specific mechanisms that remain undiscovered and thus hidden from our view. With this Research Topic, we provided a virtual space to assemble interesting studies that begin to address this critical knowledge gap and present them to readers of Frontiers of Immunology.

**Figure 1:**
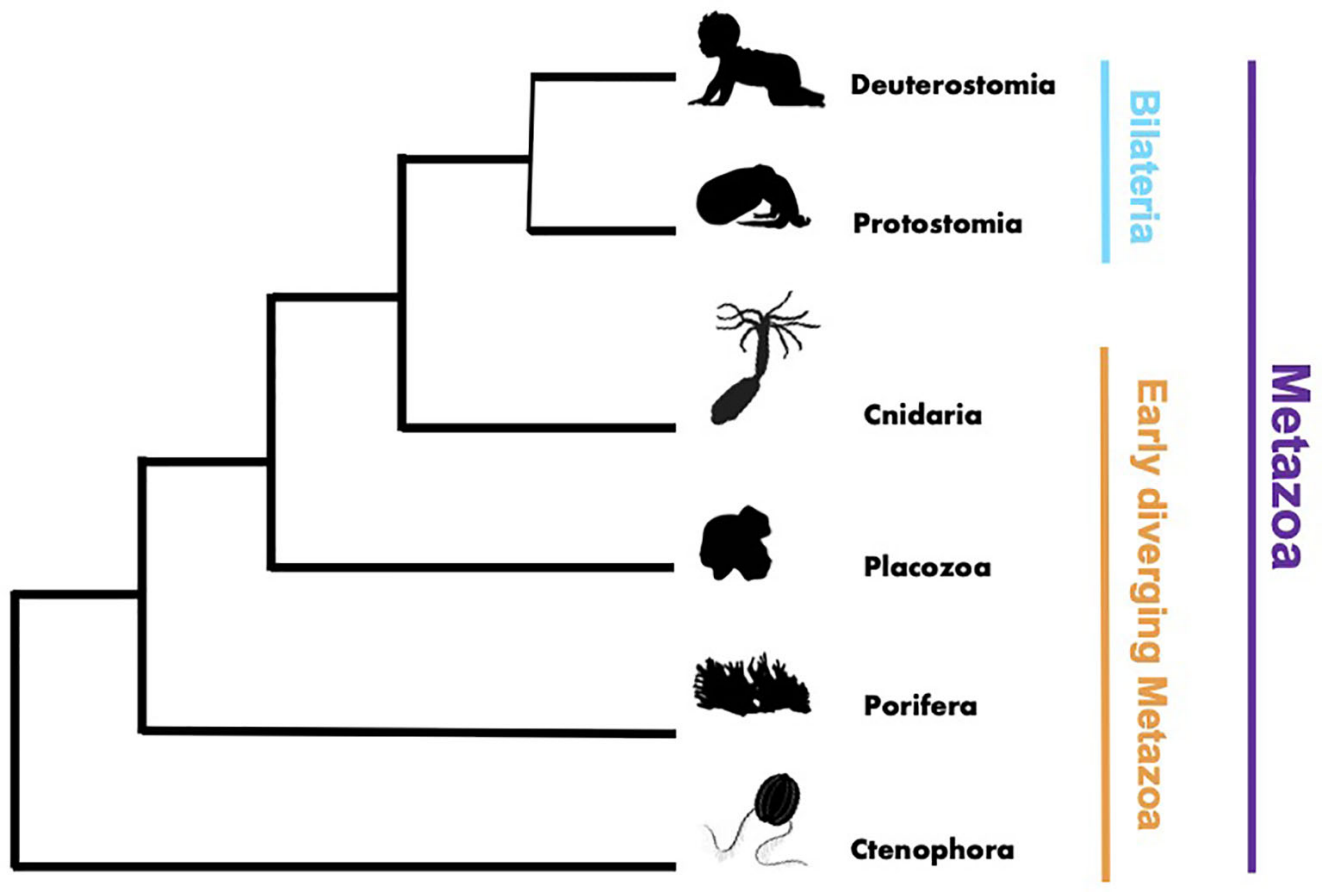
Phylogenetic tree rendering showing the broad evolutionary relationships within the Metazoa.

## Uncharted Territory: Foundational Questions Still Need Answers

Phagocytosis is a fundamental component of cellular innate immunity. The identification of specialized phagocytic immune cells in early diverging lineages, including coral, remain elusive despite the discovery of phagocytosis in the late 1800s by Élie Metschnikoff, (6) and *Drosophila* hemocytes (blood cells akin to phagocytes) studied since 1957 (7). In this issue, Snyder et al. show, for the first time, functional mechanisms associated with phagocytic cells in stony corals. Snyder et al. used fluorescence-activated cell sorting (FACS) and fluorescence microscopy to functionally identify and characterize phagocytes from the scleractinian coral, *Pocillopora damicornis*, and the actiniarian sea anemone, *Nematostella vectensis*. Their study demonstrated that phagocytes isolated from diverse anthozoan species broadly recognize and engulf targets displaying a range of microbe-associated molecular patterns (MAMPs) and danger-associated molecular patterns (DAMPs), including bacterial and fungal antigens, heat stressed self-cells, and microplastic beads. This foundational knowledge will facilitate more nuanced characterizations of immune responses in coral ecosystems sensitive to environmental perturbation. Tracey et al. demonstrated the value of understanding cellular immunity in cnidarians by using amoebocyte concentrations to monitor cellular immunity in the Caribbean sea fan octocoral, *Gorgonia ventalina*, susceptible to infection and parasitism from fungus and copepods. Amoebocyte density was used to examine immune related effects associated with environmental parameters, population demography, and host-parasite interactions. Tracey et al. found environment-specific factors and sea fan demography to be key determinants of sea fan immunity and disease amidst fungal and copepod co-infection. These two studies illustrate the importance of foundational studies, that identify and characterize components of early metazoan immunity, in addressing significant and pressing ecological questions.

## Unexpected Complexity: There Is Nuance in Early Diverging Metazoan Innate Immune Systems

Immunological complexity was a common theme within our Research Topic. For example, pathogen recognition receptors (PRRs) were identified in both Emery et al. and Schmittmann et al. Both studies showed unexpected redundancy and expansions among the evolutionarily conserved immune signaling mechanisms. Since these organisms rely solely on innate immunity to defend against pathogens, these receptor families likely play a key role in recognizing DAMPs and activating host defenses. Emery et al. identified different subphyla of Cnidaria with unique immune PRR repertoires. Emery et al. found that these unique repertoires correlated with life history traits. In sessile, colonial species, that maintained algal symbiosis expanded PRR repertoires were identified in comparison to mobile, solitary, and non-symbiotic counterparts. Schmittmann et al. found that individual sponge genotypes had unique immune gene expression profiles in response to an immune stimulus. In addition to characterizing sponge immune response involving signaling and recognition, including GTPases and post-translational regulation mechanisms like ubiquitination and phosphorylation, they were also able to connect immunity to traits such as holobiont fitness and susceptibility to stress. These studies nicely articulate the connection between immunological complexity and organismal traits that could affect survival in rapidly changing ecosystems. This theme of complexity in immune response compliments other papers in the series looking at immunity from an ecological perspective in co-infection of octocorals by Tracy et al., down to discrete cellular activity level in scleractinian corals and anemones by Snyder et al.

## Unlimited Questions: A New Dawn of Early Diverging Metazoan Immunology

Our Research Topic section in Frontiers in Immunology also presented novel areas of research in comparative immunology leading to many new and interesting questions for fruitful future investigation. For example, the immunological repertoire of other early diverging metazoans, such as ctenophores and placozoans, remain essentially unknown. Do we see the same type and range of nuanced complexity as the picture beginning to emerge from contemporary studies in Cnidaria and Porifera? In Anthozoa, how does the diverse assemblage of PRRs interact with various DAMPS? And likewise, how specific is the Anthozoa immune response tailored to a perceived pathogen? Building on current research - how are the immune systems of these organisms affected by anthropogenic climate change both regarding immediate plasticity and/or constitutive phylogenetic immune abilities that ultimately affect survival? Are we seeing functional limits in the face of quickly changing environments? How do various biological traits and environmental factors affect immunity, such as growth rate, relative reproductive state, population density, and location (in the case of sessile organisms like corals and sponges)? These and related questions represent exciting emergent areas of research that stem from this Research Topic. The topics covered in this issue have begun to functionally address fundamental questions in invertebrate comparative immunology that we hope will lead to a shift in our understanding of early diverging metazoan immunity and implications in highly unpredictable and fluctuating environments.

## Author Contributions

All authors listed have made a substantial, direct, and intellectual contribution to the work and approved it for publication.

## Funding

NT-K and BR were supported by NSF-BSF Grant: BSF-2019647, NSF-1951826. NT-K and WEB were supported by NSF-2013692. LDM: was supported by NSF OCE- 1928771 and NSF OCE-2109622. BR would like to thank Alex and Ann Lauterbach for funding the Comparative and Evolutionary Immunology Laboratory. The work of BR was supported by Israel Science Foundation (ISF) numbers: 1416/19 and 2841/19. BR has received funding from European Research Council (ERC) under the European Union’s Horizon 2020 research and innovation program under grant agreement No. 948476. BR has received funding from a HFSP grant (RGY0085/2019).

## Conflict of Interest

The authors declare that the research was conducted in the absence of any commercial or financial relationships that could be construed as a potential conflict of interest.

## Publisher’s Note

All claims expressed in this article are solely those of the authors and do not necessarily represent those of their affiliated organizations, or those of the publisher, the editors and the reviewers. Any product that may be evaluated in this article, or claim that may be made by its manufacturer, is not guaranteed or endorsed by the publisher.
